# High Bee and Wasp Diversity in a Heterogeneous Tropical Farming System Compared to Protected Forest

**DOI:** 10.1371/journal.pone.0052109

**Published:** 2012-12-26

**Authors:** Christof Schüepp, Sarah Rittiner, Martin H. Entling

**Affiliations:** 1 Institute of Ecology and Evolution, Community Ecology, University of Bern, Bern, Switzerland; 2 University of Koblenz-Landau, Ecosystem Analysis, Landau, Germany; University of Tartu, Estonia

## Abstract

It is a globally important challenge to meet increasing demands for resources and, at the same time, protect biodiversity and ecosystem services. Farming is usually regarded as a major threat to biodiversity due to its expansion into natural areas. We compared biodiversity of bees and wasps between heterogeneous small-scale farming areas and protected forest in northern coastal Belize, Central America. Malaise traps operated for three months during the transition from wet to dry season. Farming areas consisted of a mosaic of mixed crop types, open habitat, secondary forest, and agroforestry. Mean species richness per site (alpha diversity), as well as spatial and temporal community variation (beta diversity) of bees and wasps were equal or higher in farming areas compared to protected forest. The higher species richness and community variation in farmland was due to additional species that did not occur in the forest, whereas most species trapped in forest were also found in farming areas. The overall regional species richness (gamma diversity) increased by 70% with the inclusion of farming areas. Our results suggest that small-scale farming systems adjacent to protected forest may not only conserve, but even favour, biodiversity of some taxonomic groups. We can, however, not exclude possible declines of bee and wasp diversity in more intensified farmland or in landscapes completely covered by heterogeneous farming systems.

## Introduction

### Importance of Modified Land for Biodiversity Conservation

Although a considerable amount (>13%) of the world’s terrestrial surface is nowadays designated as nationally or internationally protected areas [1] the effectiveness of protected areas in biodiversity conservation is limited, especially in the tropics: First, financial support for basic management activities to stop illegal and harmful human activities within parks is often lacking [2]. Second, about 12% of terrestrial vertebrates, mostly endemics, are not covered within the current protected area network [3]. And third, the long-term effectiveness of reserves depends strongly on human population density and activities in the surroundings [4]. Thus, more recently research on biodiversity conservation in the tropics has moved away from strictly focussing on protected areas [5] to considering also the importance of anthropogenic habitats within a landscape mosaic [6]–[9].This change of focus is crucial when taking into account increased competition for land, due to both increasing demands of resources (caused by human demography and wealth) and, at the same time, increasing loss of agricultural land due to climate change and urbanisation [10].

### Effects of Human Land-Modifications on Biodiversity

The ‘intermediate disturbance hypothesis’ postulates maximum diversity at intermediate regimes of disturbance [11]. Although the validity of the underlying mechanisms is currently discussed [12], this hypothesis has been widely tested in marine [13] and forest ecosystems [14], and can be applied to agricultural systems [15]. Increased anthropogenic disturbance in agricultural landscapes generally leads to declines in species diversity [8], [16]–[19]. However, if land modification is moderate and leads to a more heterogeneous landscape it can also increase biodiversity [8], [20]–[23]. It remains an important question to what extent natural habitats can be modified without decreasing diversity [24], [25] or, rephrased, how many species from natural habitats are retained in modified habitats with a certain level of disturbance [7]. In this study, we investigated a small-scale farming system with heterogeneous land-use practices embedded in a tropical forest landscape.

### Spatial and Temporal Community Variation (Beta Diversity)

Spatial heterogeneity within one habitat type can lead to high variation of community composition between study sites [26]–[28]. If spatial heterogeneity is higher in a natural compared to a modified habitat, focussing on mean species richness per site can lead to serious underestimation of the conservation value of the more natural habitat. Therefore, several authors emphasize the value of reporting community variation (beta diversity) over the whole study area [17], [25], [27], [29]. Furthermore, habitat loss and land-use change can lead to the dominance of disturbance-tolerant generalist species [30] and thus increase the similarity of communities (effects on bees [31], on plants [32], on bees and wasps [33]). The same is true for temporal heterogeneity: Strong temporal heterogeneity in biodiversity of a more natural habitat can lead to a serious underestimation of its conservation value, if sampling is temporally restricted to one season [33], [34]. In this study, we collected insects over a spatiotemporal climatic gradient and included beta diversity in our analysis (see ‘Materials and Methods’).

### Conservation Research on Arthropods

Despite their overriding diversity and their importance for humankind through providing ecosystem services such as pollination or pest control [35]–[37], arthropods are widely neglected in conservation policies [38]. However, conservation efforts based on other taxa, such as plant or vertebrates, are often inappropriate for arthropods because of low cross-taxon congruence [6], [7], [27], [31], [39]. Effective conservation science therefore needs to incorporate more arthropod research [25]. Within arthropods, responses to habitat modification may depend strongly on life-history traits of species. Predators and parasitoids of higher trophic levels may be more strongly affected by land modification than their hosts or prey [40]–[44]. Therefore, we investigated three trophic groups of bees and wasps (Insecta: Hymenoptera): Bees (Apidae, Colletidae, Halictidae, Megachilidae) are important pollinators of wild and crop plants [35], [36]. Paper wasps (Vespidae) predate on insects and play a role in biological pest control [45]. Spider wasps (Pompilidae) are secondary carnivores (fourth to higher trophic level) by using spiders as larval food [45]. Besides trophic level, degree of sociality and nesting requirements in arthropods may influence the susceptibility to habitat modification [46]–[50]. If species with different life-history traits are not analysed separately, effects of land modification may be masked by opposite reactions. Here, we analysed separately the three trophic groups mentioned above and controlled for opposing effects of different degrees of sociality and nesting requirements in bees.

### Hypotheses

The main goal of this study was to quantify the impact of farming areas on biodiversity. In contrast to previous studies, we selected farming areas embedded in a landscape with a high proportion of protected forest and low level of anthropogenic disturbance. We focussed on three groups of bees and wasps at different trophic levels, of which two (paper and spider wasps) are very rarely used in biodiversity assessments, and for which human impact through land modification is basically unknown. Because farming areas were relatively small and heterogeneous in their land-use practices, we expected higher diversity (richness, abundance and evenness) of bees and wasps in farming areas. We further tested if different degrees of sociality and nesting requirements in bees lead to different responses to land modification. Finally, we hypothesized a higher spatiotemporal community variation (beta diversity) in forest compared to farmland because of naturally occurring heterogeneity between forest sites in the northeast and southwest of our study region (changes in forest structure and plant community; see section ‘Study Area’ in ‘Materials and Methods’).

## Materials and Methods

### Ethics Statement

Insects were collected and exported under the Belizean Forest Department research permit no. CD/60/3/09 (35), issued on November 9, 2009. Additionally, we got oral or written approvals for insect sampling from all private land owners (eight farmers and two owners of private protected areas). Bees and wasps are not legally protected in Belize.

### Study Area

The study was conducted in northern coastal Belize, Central America, south and west of the village Sarteneja in Corozal District (N 18°12′-18°20′/W 88°07′-88°16′). The study sites were spread over an area of about 400 km^2^ and along a climatic gradient from dry and hot ‘Yucatecan low semi-deciduous forest’ in the northeast to cooler and more humid ‘Yucatecan medium-sized semi-evergreen forest’ and ‘*Cohune*-palm dominated forest’ in the southwest [51]. All forest types were relatively dry, low in stature, and seasonal as compared to most of Central America. Besides forest, the main habitat types of the area were savannah, brackish water lagoons, mangroves, some human settlements, and small-scale farming areas. Mean annual rainfall ranged from approx. 1500 mm in the north to 2000 mm in the west and south [52] and mean temperature during the study period differed between 24.3°C in the north and 22.3°C in the west and south (measured using DS1923 Hygrochron iButtons, Maxim, Sunnyvale, USA).

### Study Sites

Both the study sites in the forest (seven sites) and in small scale farming areas (eight sites) were equally distributed over the entire study area and along the whole climatic gradient of the study area (Fig. S1). Forest and farming sites were interspersed to avoid problems of spatial autocorrelation. Five out of the seven study sites in forest lay within the protected area of Shipstern Nature Reserve (www.shipstern.org), one lay within a smaller private reserve (www.wildtracksbelize.org), and one in an unused forest close to the private land of the same organisation. Although forest sites were under protection for many years, in the past all of them were subject to some human disturbance (possibly including selective logging) and natural disturbance (hurricanes). Therefore we use the term ‘natural’ or ‘protected forest’ instead of ‘primary forest’. Forest sites were located between 120 and 140 m from the next forest edge of farmland or clearings for forest roads. Farming areas consisted of a mosaic of mixed crop types, open land, agroforestry, and secondary forest. Within the vicinity of traps in farmland, we monitored more than 30 species of crops. Most crops are grown for subsistence. However, some cash crops (e.g. plantain, banana, bean, and onions) are sold to local markets, and recently Mahogany trees were planted to sell to the international timber market. Farmland size ranged from 3–80 ha and conversion from forest into farmland took place 8–70 years ago. All farming areas were fully surrounded by natural forest. Traps in farming areas were set at least 100 m from the next natural forest edge. The mean distance between two study sites was 9.9 km (min: 440 m, max: 17 km). The minimum distance between two study sites of the same habitat type was 1.3 km.

### Trapping Methods

One Malaise trap (B&S Entomological Services, UK) was installed at each study site. Malaise traps are an efficient method for sampling flying insects [53] and also adequate in tropical forest systems [54]. Traps operated from December 2009 to February 2010, covering both the wet season with heavy rain falls in December and the beginning of the dry season in February. All traps were sampled weekly. Every second week a small container (6×5×5 cm) with orchid scents was hung into the Malaise trap to attract male orchid bees (Apidae: Euglossini), which are otherwise rarely collected [55]. Male orchid bees gather chemical compounds from orchids and other plant families and display them during courtship [55]. We used three of the most commonly used attractants: cineole, eugenol (each 0.4 ml/week), and methyl salicylate (0.8 ml/week). Scents constantly dispersed over one week through four small holes in the container (2 mm diameter each). Although, to our knowledge, negative (repellent) effects of attractants on non-orchid bee species has never been reported, we added scents to traps only every second week to avoid possible exclusion of some sensitive bee species. Whereas the presence of attractants had a very strong positive effect (almost hundredfold increase) on abundance of orchid bees (linear mixed effect model with scent as fixed and study sites as random factor: p<0.001) it had no effect on the abundance of non-orchid bees (same model: p = 0.17). Because trapping efficiency of Malaise traps depend on vegetation structure the traps in farming areas were set up in locations characterised by understory vegetation and a closed canopy to mimic the same vegetation structure as in forest.

### Determination of Bees and Wasps

Collected bees and wasps were either mounted (bees and spider wasps) or stored in 85% ethanol (paper wasps), and identified by specialists (see Acknowledgements). More than 88% of all individuals were determined to species level (72 species). The remaining samples were assigned to morphospecies (36 morphospecies) because revisions of many neotropic bee and wasp genera are still lacking. Hereafter, morphospecies and species are referred to simply as *species*. Bees were assigned to four groups of sociality (solitary, social, eusocial, and cleptoparasitic) and three group of nesting requirements (cavity-nesting, ground-nesting, and wood-nesting) [45], [49], [56], [57] (Table S1). ‘Social’ comprised all live forms between strictly solitary and eusocial, namely communal, semisocial, and primitively eusocial. Bees in the genera *Augochlora*, *Ceratina*, *Euglossa*, and *Lasioglossum* were classified as social, although this is not certain for every species. Nesting of cleptoparasitic bees was defined according to the nests of their hosts. All collected specimens, except some vouchers, are archived in the Natural History Museum of Bern, Switzerland (Naturhistorisches Museum der Burgergemeinde Bern).

### Statistical Analysis

Differences in species richness and abundance between forest and farming areas were analysed using Generalized Linear Models (GLM) for overdispersed count data (quasi-poisson errors with a log link function), and differences in Simpson’s Evenness E_1/D_ [58] were analysed using non-parametric Wilcoxon rank sum test because residuals could not be normalised with any transformation. Because the observed number of species is sensitive to the number of individuals sampled [59] we performed individual-based rarefaction curves, using the open source software EstimateS 8.2.0 [60]. Forest and farmland were compared using sample-based rarefaction curves, where individuals were set as samples [6]. Rarefaction curves and 95% confidence intervals were calculated using Mao Tau estimator. Significance (at p<0.05) was inferred if the total observed richness of the habitat with the smaller sample (habitat type with lower number of individuals) fell outside the confidence interval of the larger sample.

To determine temporal community variation (temporal beta diversity) we performed additive partitioning of species diversity [61], using spatial replication (study sites) and temporal variation (weeks) [33]. Two major criticisms exist against additive partitioning: First, beta diversity is not independent of alpha, thus formulating a problem if alpha diversities of compared habitats are very different [62]. Second, the measure of additive beta diversity loses its resolution for datasets with many samples sharing few species [63]. We consider these limitations of minor importance for the current study, because alpha diversity of forest and farmland were within the same order of magnitude and because a considerable number of species were shared between samples. Because in additive partitioning spatial beta diversity is not replicated and therefore not testable, we instead compared homogeneity of multivariate dispersions between forest and farmland based on Sørensen (presence/absence) and Bray-Curtis (abundance included) dissimilarities (Method V4 in [63]). Differences in temporal species variation (additive partitioning) and spatial homogeneity of multivariate dispersions between forest and farmland were tested using Generalised Least Squares Models (GLS), taking into account heterogeneity of residual variances [64]. Diagnostic plots of GLS indicated normal distribution of residuals. Apart from rarefaction curves, all analyses were carried out in the open source software R 2.12.2 [65], using the packages vegan [66] and nlme [67].

## Results

### Local (Alpha) Diversity

**Figure 1:**
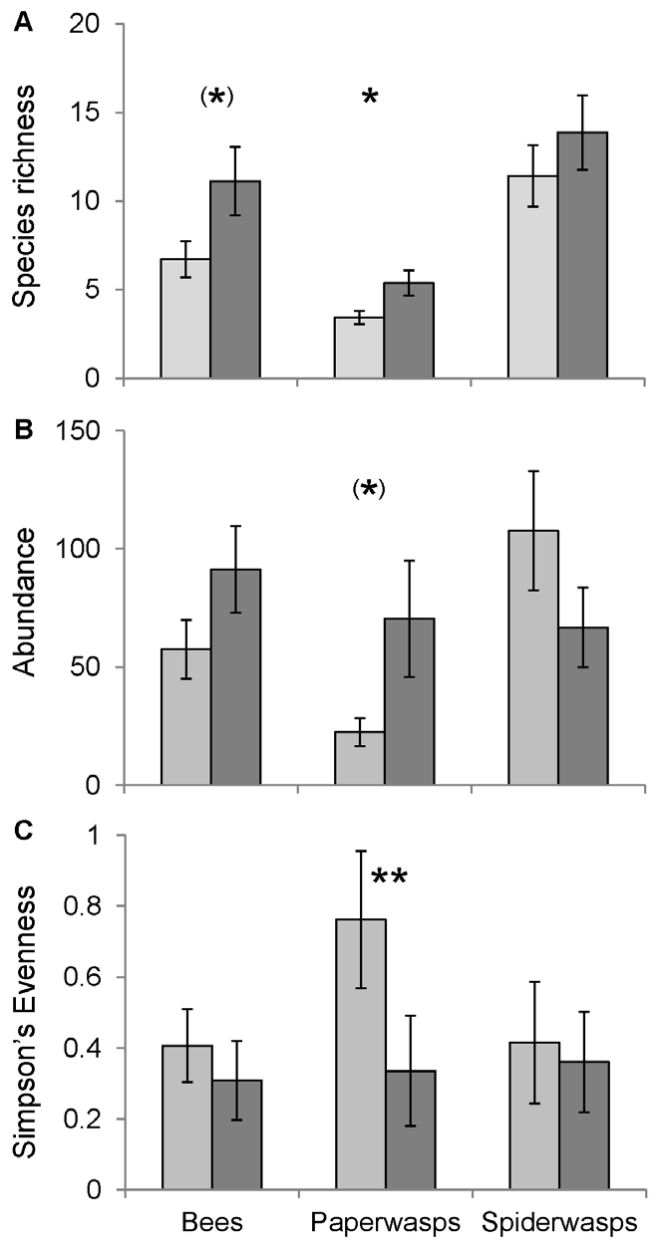
Biodiversity of bees and wasps. Differences in (A) species richness (mean no. of species per study site), (B) abundance (mean no. of collected individuals per site), and (C) Simpson’s Evenness per site for bees, paper wasps, and spider wasps between protected forest (light grey) and heterogeneous farmland (dark grey). Error bars show standard error of the mean. Significance levels: ** p<0.01, * p<0.05, ^(^*^)^ p<0.1.

**Figure 2:**
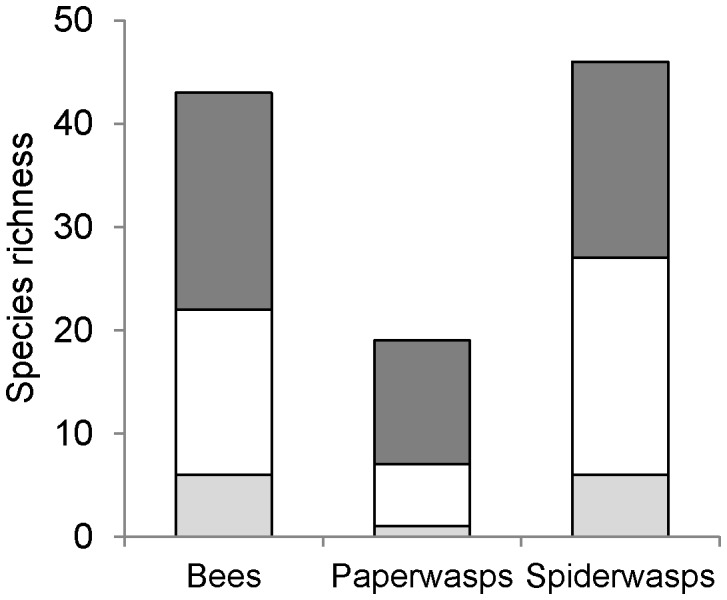
Exclusive and shared species across habitat types. Pooled species richness (over 3 months and 15 study sites) of bees, paper wasps, and spider wasps exclusively trapped in protected forest (light grey), in heterogeneous farmland (dark grey), and trapped in both habitat types (white).

**Figure 3:**
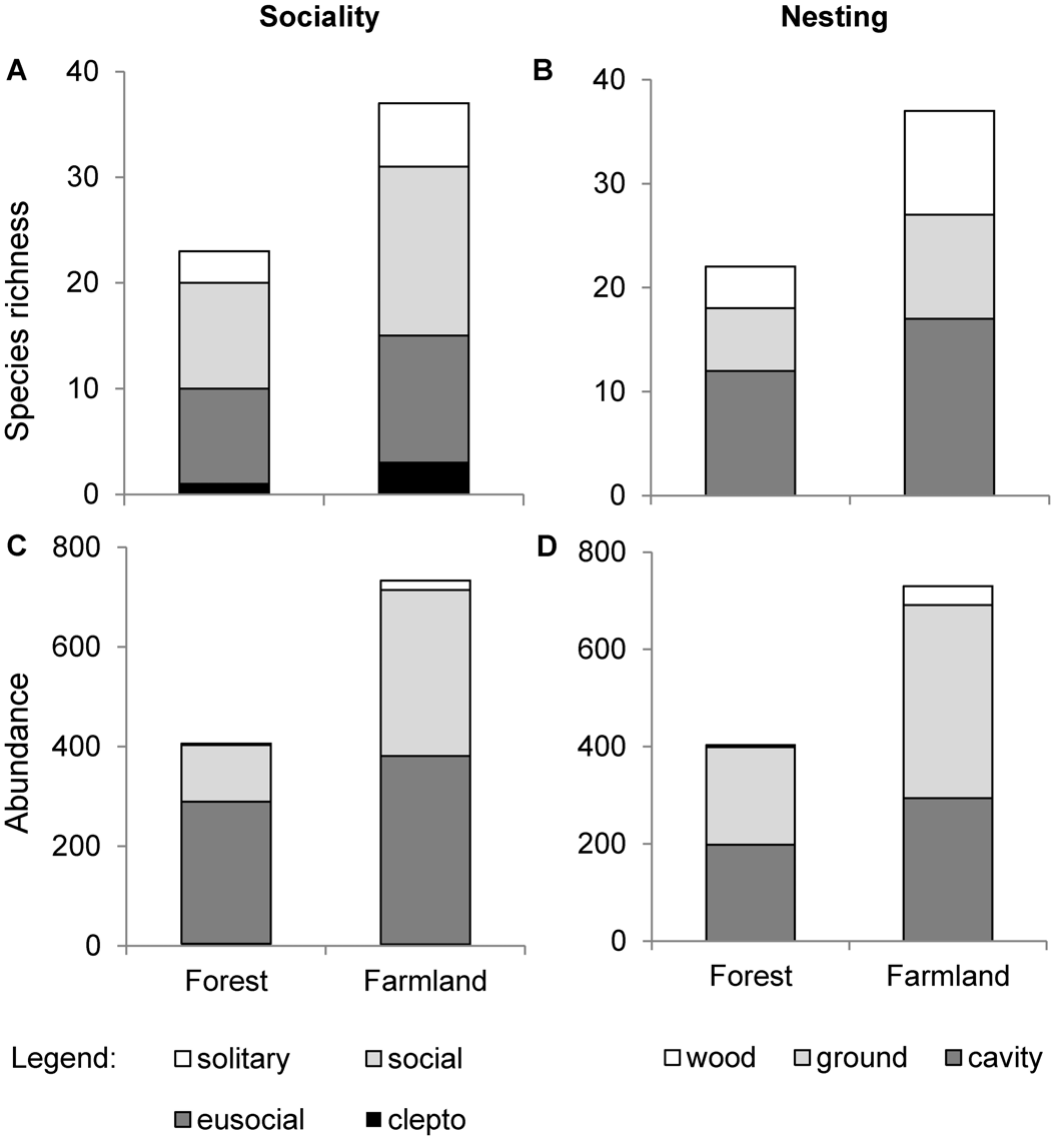
Degree of sociality and nesting requirements **in bees.** Differences in (A, B) species richness and (C, D) abundance of bees between protected forest and heterogeneous farmland (pooled data over 3 months and 15 study sites). Bees are separated into four degrees of sociality (solitary, social, eusocial, and cleptoparasitic) and three groups of nesting requirements (wood-nesting, ground-nesting, cavity nesting). For assignment of bees see Table S1.

**Figure 4:**
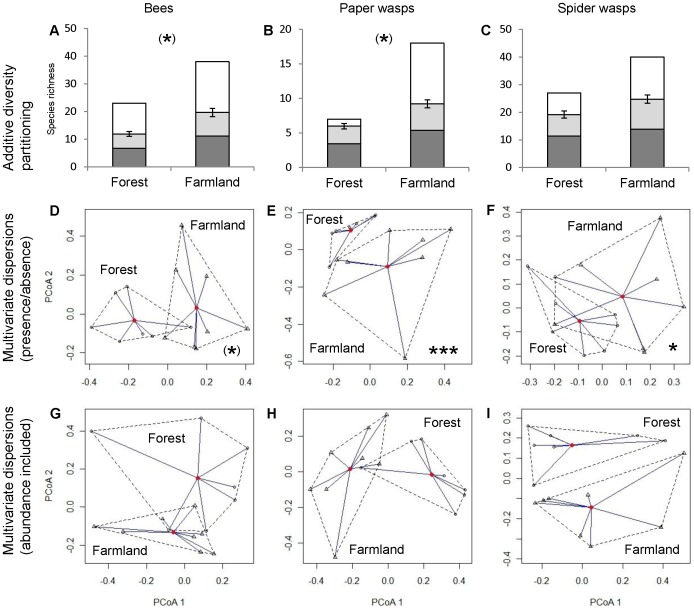
Community variation (beta diversity) of bees and wasps. (A–C) Alpha diversity (dark grey), temporal beta diversity (light grey), and spatial beta diversity (white) compared between protected forest and heterogeneous farmland based on additive diversity partitioning. Temporal beta diversity was replicated and statistically tested. Error bars show standard error of the mean. Total height of bars indicates overall (gamma) diversity within forest or farmland. (D–I) Spatial beta diversity, measured as distance to centroid (homogeneity of multivariate dispersions), was based on (D–F) Sørensen dissimilarities (presence/absence) and (G–I) Bray-Curtis dissimilarities (abundance included). Points (forest) and triangles (farmland) represent species composition of one study site. Larger distances between study sites and the mean composition of a habitat type (unbroken lines) indicate higher difference in species composition. Dashed lines connect all sites of one habitat type. Significance levels: *** p<0.001, * p<0.05, ^(^*^)^ p<0.1.

In total, 1133 bees of 43 species, 720 paper wasps of 19 species and 1288 spider wasps of 46 species were collected (Table S1). Species richness of paper wasps was higher in farming areas compared to protected forest (t_1,13_ = 2.43; p = 0.030) and bees showed a trend in the same direction (t_1,13_ = 2.01; p = 0.066) (Fig. 1A). Species richness of spider wasps was not significantly different between farming areas and forest (t_1,13_ = 0.88; p = 0.40). The higher species richness in farming areas was largely due to species trapped in farmland that did not occur in the forest. Conversely, a high percentage of species trapped in forest was also found in farming areas (72% of bees, 85% of paper wasps, and 77% of spider wasps) (Fig. 2). Abundance did not differ significantly between forest and farmland in any of the three groups (bees: t_1,13_ = 1.50; p = 0.16; paper wasps: t_1,13_ = 1.95; p = 0.073; spider wasps: t_1,13_ = −1.39; p = 0.19; Fig. 1B). Simpson’s evenness of paper wasps was significantly higher in farming areas than in forest (W_7,8_ = 53; p = 0.002), but bees and spider wasps did not differ between habitat types (bees: W_7,8_ = 40; p = 0.19; spider wasps: W_7,8_ = 34; p = 0.54; Fig. 1C). Rarefaction curves show higher species richness in farmland for bees and spider wasps but not for paper wasps (Fig. S2). Bees showed consistently higher species richness and abundance in farming areas compared to forest in all degrees of sociality and nesting requirements (Fig. 3). Overall species richness (gamma diversity) was 70% higher in farmland (Fig. 4A–C, gamma diversity indicated by total height of bars).

### Community Variation (Beta Diversity)

Bees and paper wasps showed a trend towards higher temporal community variation (beta diversity) in farmland compared to forest (bees: t_1,13_ = 1.96; p = 0.072; paper wasps: t_1,13_ = 1.85; p = 0.087; Fig. 4A,B). Temporal community variation of spider wasps did not show a significant pattern (t_1,13_ = 1.56; p = 0.139; Fig. 4C). Spatial community variation (beta diversity) based on presence/absence data (Sørensen) was significantly higher in farmland for wasps (paper wasps: t_1,13_ = 5.40; p<0.001; spider wasps: t_1,13_ = 2.67; p = 0.019; Fig. 4E,F) but this trend was not statistically significant for bees (t_1,13_ = 1.87; p = 0.085; Fig. 4D). Spatial community variation (beta diversity) based on abundance data (Bray-Curtis) did not differ between forest and farmland in any of the three taxa (|t_1,13_|<1.15; p>0.269; Fig. 4G–I).

## Discussion

### Local (Alpha) Diversity

Our results based on linear models and rarefaction curves suggest that small-scale farming systems do not only conserve but even favour biodiversity of some taxonomic groups. Species richness of bees and wasps was higher in farmland areas, first because most species occurring in adjacent forest were also found within farmland, and second because many exclusive species inhabited farmland that were not trapped in forest (Fig. 2). In other words, the proportion of species trapped in forest inhabiting or using farmland was high, indicating the compatibility of this farming system with bee and wasp conservation [7]. The studied farmland type matches perfectly the description of favourable agriculture for pollinators by Kremen [68]: ”Positive effects of agriculture on pollinator communities may be more likely to occur in regions in which the presence of agriculture increases habitat heterogeneity, such as farming landscapes that include relatively small field sizes, mixed crop types within and between fields, and patches of noncrop vegetation, such as hedgerows, fallow field, meadows, and seminatural wood or shrublands”. In our study region, a natural forest matrix surrounded the small-scale farming areas within dispersal and foraging distance of investigated bees and wasps. Because distance to natural habitat strongly decreases richness and abundance of bees [18], [50] it remains unclear to what extent species trapped in forest may maintain viable populations in landscapes covered completely by these farmland habitats [9]. Even if bee and wasp species richness was equal or higher within farmland, and most species trapped in forests were also found in farming areas, forest may nevertheless be a vital component within the life cycle of some or many of the species and may serve as constant source for colonisation of farmland. In accordance with our results, two studies on plants, vertebrates and invertebrates showed that a considerable number of forest species persist in secondary forest, agroforestry, and even pastures if the surrounding landscape comprises high amounts of mature forest [69], [70].

In addition to the desirable features of the studied agricultural system, bees and wasps in tropical forest may be less sensitive to human disturbance compared to other taxa [6], [27]. This emphasises that our results cannot be generalised to other taxa. At least, in our study effects of land modification were more or less consistent between investigated groups of bees and wasps. The same was true in a study on bees and wasps in Ecuador [71]. Furthermore, all groups of sociality and nesting requirements in bees were positively affected by land modification (Fig. 3), in contrast to previous findings where effects of habitat types or levels of disturbance on bee communities depended on life history traits [46], [47], [49].

### Community Variation (Beta Diversity)

In contrast to our hypothesis, spatial community variation (beta diversity) of bees and wasps was equal or higher in farming areas compared to forest (Fig. 4A–F). High beta diversity within a study region can be a statistical artefact due to low local alpha diversity [72]. However, in our study we can exclude such an inflation of beta diversity because both alpha and beta diversity were increased in farmland. Habitat heterogeneity is a major driver of community variation [26]–[28]. Thus, differences in farming areas due to variable land-use practices could have led to higher beta diversity in farmland. High management diversity between different agroforestry plots within a study region appeared to be responsible for high beta diversity of bees in Indonesia [73]. Spatial community variation disappeared when analyses were performed including abundances (Fig. 4G–I), showing that effects of beta diversity were driven by the high percentage of species with very low abundances (29% of species are singletons).

Temporal community variation of bees and paper wasps tended to be higher in farmland than in forest, in contrast to a study in Ecuador [33] where lower temporal beta diversity was found in more disturbed habitat types. However, in that study lower beta diversity occurred only in open habitat types, whereas we compared protected forest against a farming system consisting of a considerable amount of woody habitat. In conclusion, the increase in the regional species pool (gamma diversity) by 70% through species only occurring in farming areas (Fig. 4A–C) was composed of both, higher local diversity (alpha) and higher spatial and temporal community variation (beta diversity).

### Effects of Human Disturbance on Bees and Wasps

We found that species richness of bees and wasps was favoured in farmland areas compared to protected forest and we conclude that bees and wasps occurring in natural forest were not susceptible to the extent and disturbance level of the studied farming system [7]. Our results are in line with the ‘intermediate disturbance hypothesis’ [11] predicting higher biodiversity at higher levels of disturbance in generally undisturbed, relatively natural landscapes. Farming areas create additional habitats and benefit biodiversity through increased heterogeneity of resources within foraging distance [8], [22], [49], [74], [75]. To the extreme, disturbed areas may even lead to a spillover of rich farmland bee communities into forest remnants [76], [77]. However, this may not necessarily be beneficial considering the competitive advantageous of some abundant generalist farmland species. The resilience of forest bees to disturbance may hold as long as some elements of native habitats remain in the disturbed areas [47], [78]. However, in very intensively managed landscapes, additional disturbance leads to a reduction of diversity [8], [16]. Due to the presence of naturally occurring disturbance in Belizean forests caused by hurricanes, forest communities may be less sensitive to disturbance by logging, as proposed for butterflies by Lewis [79].

From a conservation perspective, it is debatable if species inhabiting modified habitats but not occurring in native forest are of high concern. Disturbed areas may be first invaded by disturbance-tolerant widespread or even non-native generalist species, which are of low conservation value [6], [23]. These species may become dominant components in communities of disturbed habitats. In Britain, continuing habitat degradation led to an increased dominance of generalist butterfly species [30]. The lower Simpson’s Evenness of paper wasps in farmland compared to forest reflects the high dominance [80] of the most abundant species in farming areas, namely *Polybia occidentalis nigratella* (Table S1). However, this species is also the most abundant species in forest and it is still an open research question how important community evenness is for ecosystem functioning [17]. Comprehensive comparisons of the biogeography and conservation value of species between farming areas and forest are not possible in our study due to lack of sound information on species distributions, rarity, and specialisation, and because some individuals could only be determined to morphospecies.

### Conclusions

Our results show that agricultural systems exist, where local (alpha) diversity and variation in communities (beta diversity) are similar or higher than in protected forest and where species occurring in forests also use these farmlands. We conclude that heterogeneous and small-scale farming areas embedded in a matrix of protected forest are compatible with biodiversity conservation for some taxonomic groups. However, it remains unclear to what extent these systems would retain forest species if protected forest in the surrounding was further reduced or if existing farmland areas are further intensified. Finally, we do not conclude that a transfer of forest into heterogeneous small-scale farming systems is desirable. But, if necessary to meet demands of local populations, it can be tolerated from the perspective of bee and wasp conservation.

## Supporting Information

Figure S1 **Study area.** Google Earth [81] map showing the study area in northern Belize, Central America. Study sites in heterogeneous farmland are marked in red, sites in protected forest in yellow.
(KML)

Figure S2 **Individual-based rarefaction curves.** Solid lines are rarefaction curves for (A) bees, (B) paper wasps, and (C) spider wasps in protected forest (grey) and heterogeneous farmland (black) and dotted lines are 95% confidence intervals for the larger sample, i.e. the habitat type with the higher total amount of individuals (see ‘Materials and Methods’ for details).
(PDF)

Table S1 Species list. Bees, paper wasps, and spider wasps with total abundances in protected forest and heterogeneous farmland. Bees are assigned to different life-history traits (LHT).
(PDF)
