# Supplementary material for: High Bee and Wasp Diversity in a Heterogeneous Tropical Farming System Compared to Protected Forest
Source: PLoS One. 2012 Dec 26;7(12):e52109. doi: 10.1371/journal.pone.0052109 (PMC3530594; doi:10.1371/journal.pone.0052109)

Supporting Information

Schüepp et al.: High bee and wasp diversity in a heterogeneous tropical farming system compared to protected forest.

Figure S2. Individual-based rarefaction curves (solid lines) with 95% confidence interval for the larger sample (dotted lines). See ‘Materials and Methods’ for details.

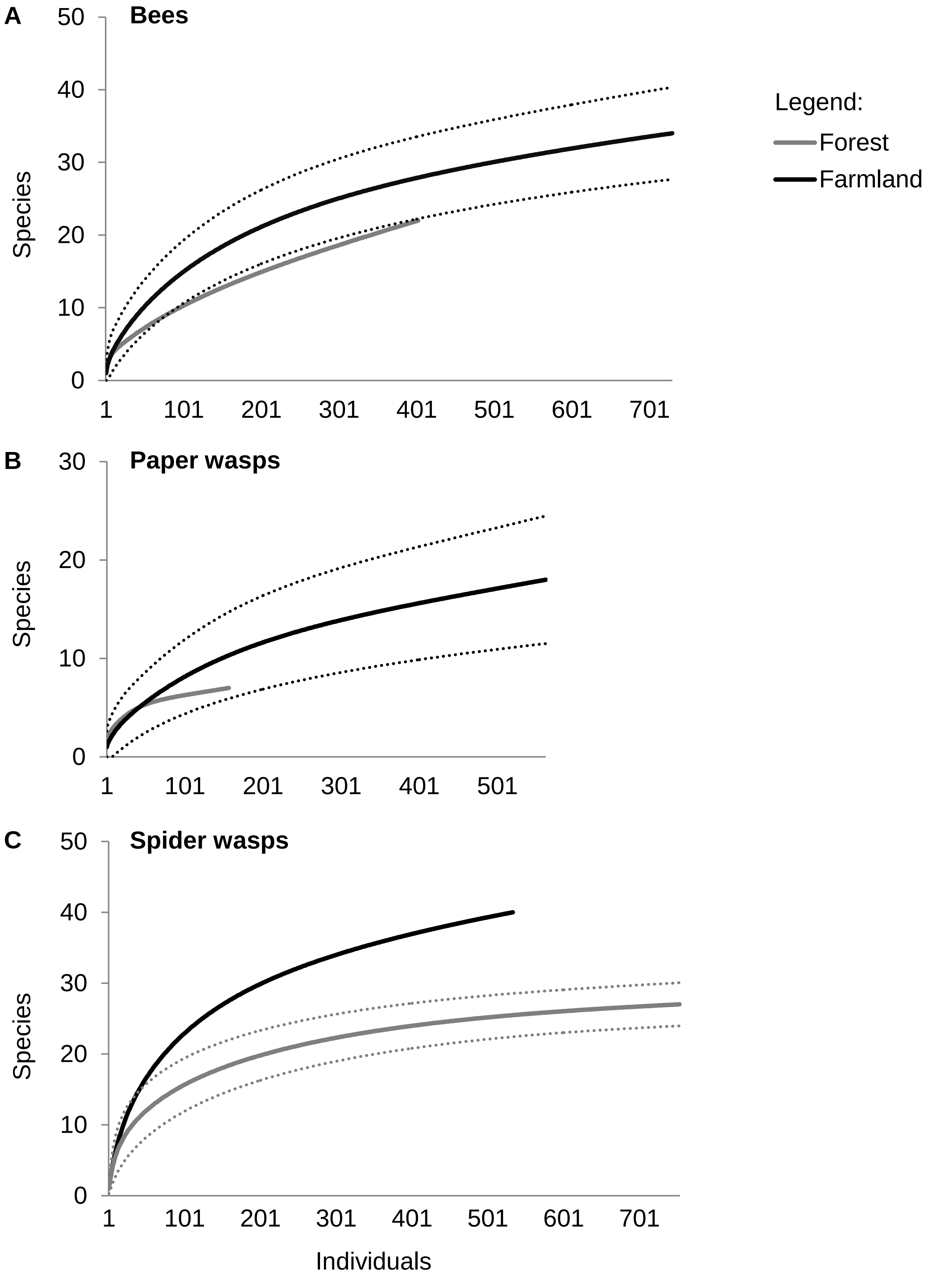

Supplement: Figure S2 — Individual-based rarefaction curves. Solid lines are rarefaction curves for (A) bees, (B) paper wasps, and (C) spider wasps in protected forest (grey) and heterogeneous farmland (black) and dotted lines are 95% confidence intervals for the larger sample, i.e. the habitat type with the higher total amount of individuals (see ‘Materials and Methods’ for details). (PDF) [file pone.0052109.s002.pdf]
