# Supplementary material for: High Bee and Wasp Diversity in a Heterogeneous Tropical Farming System Compared to Protected Forest
Source: PLoS One. 2012 Dec 26;7(12):e52109. doi: 10.1371/journal.pone.0052109 (PMC3530594; doi:10.1371/journal.pone.0052109)
Supplement: Table S1 — Species list. Bees, paper wasps, and spider wasps with total abundances in protected forest and heterogeneous farmland. Bees are assigned to different life-history traits (LHT). (PDF) [file pone.0052109.s003.pdf]

Supporting Information

Schüepp et al.: High bee and wasp diversity in a heterogeneous tropical farming system compared to protected forest.

Table S1. Species list with total abundances in forest (n=7) and farming areas (n=8).

Family	Tribe	Genus	Species	LHT[1]	Forest	Farmland
Apidae	Apini	*Apis*	*mellifera* Linnaeus, 1758	eus, c	1	24
	Ceratinini	*Ceratina* (*Calloceratina*)	cf *regalis* Cockerell, 1912	soc, w		7
			eximia Smith, 1862	soc, w		4
			viridicincta Cockerell, 1931	soc, w		5
		Ceratina (*Zadontomerus*)	sp.	soc, w		7
		Ceratina (*Ceratinula*)	sp.	soc, w	1	
	Epeolini	*Triepeolus*	sp.	cle, g		1
	Eucerini	*Peponapis* (*Peponapis*)	cf *limitaris* (Cockerell, 1906)	sol, g	1	5
	Euglossini	*Euglossa*	*tridentata* Moure, 1970	soc, c(e)		1
			variabilis Friese, 1899	soc, c(e)	1	6
			viridissima Friese, 1900 [2]	soc, c	91	222
		Exaerete	*smaragdina* (Guérin-M., 1844)	cle, c(e)	4	
	Exomalopsini	*Exomalopsis*	sp.	soc, g		1
	Meliponini	*Cephalotrigona*	*zexmeniae* (Cockerell, 1912)	eus, c		1
		Frieseomelitta	*nigra* (Cresson, 1878)	eus, c		1
		Lestrimelitta	*niitkib* Ayala, 1999	eus, c	4	1
		Melipona	*beecheii* Bennett, 1831	eus, c	1	9
		Nannotrigona	*perilampoides* (Cresson, 1878)	eus, c		1
		Partamona (*Partamona*)	*bilineata* (Say, 1837)	eus, c	2	5
		Plebeia	cf *frontalis* (Friese, 1911)	eus, c	87	8
		Scaptotrigona	*pectoralis* (Dalla Torre, 1896)	eus, c		1
		Trigona	*corvina* Cockerell, 1913	eus, c(e)	1	
			fulviventris Guérin-M., 1844	eus, g	184	323
			fuscipennis Friese, 1900	eus, c	1	2
		Trigonisca	sp.	eus, c	4	2
	Osirini	*Osiris*	sp.	cle, w		1
	Tapinotaspidini	*Paratetrapedia* (*Lophopedia*)	sp.	sol, w	1	
		Paratetrapedia	sp.	sol, w		2
Colletidae	Caupolicanini	*Ptiloglossa*	sp. A	sol, g		3
			sp. B	sol, g		1
Halictidae	Augochlorini	*Augochlora* (*Augochlora*)	cf. *nigrocyanea* Cockerell, 1897	soc, w		5
			sp. B	soc, w	1	
			sp. C	soc, w	1	5
			sp. D	soc, w		1
		Augochlora (*Oxystoglossella*)	sp.	soc, g	1	14
		Augochloropsis (*Paraugo*.)	sp.	soc, g	1	3
		Caenaugochlora (*Caenaugo*.)	sp.	soc, g	3	
		Megalopta	cf *centralis* Friese, 1926	soc, w		2
		Pereirapis	*semiaurata* (Spinola, 1851)	soc, g		4
	Halictini	*Lasioglossum* (*Dialictus*)	sp.	soc, g	11	42
Megachilidae	Megachilini	*Coelioxys* (*Cyrtocoelioxys*)	sp.	cle, c(e)		1
		Megachile (*Chelostomoides*)	sp.	sol, c(e)		7
		Megachile (*Leptorachis*)	sp.	sol, c(e)	1	2

[1] Life-history traits (LHT) of bees: eus = eusocial, soc = social, sol = solitary, cle = cleptoparasitic, c = cavity-nesting, g = ground-nesting, w = wood-nesting, (e) = potentially also building exposed nests. *Augochlora*, *Ceratina*, *Euglossa*, and *Lasioglossum* were classified as social, although this is not certain for every species. Nesting of cleptoparasitic bees was defined according to the nests of their hosts.

[2] potentially including cryptic sibling species *E. dilemma* Bembé & Eltz 2011

Table S1 (cont.)

Family	Subfamily	Genus	Species	Forest	Farmland
Pompilidae	Ceropalinae	*Ceropales*	*cubensis albopicta* Cresson, 1865		8
			rugata Townes, 1957		2
		Irenangelus	*hispaniolae* Evans, 1969	4	
	Pepsinae	*Ageniella*	*fabricii* Banks, 1944		2
			maya Banks, 1931	2	5
			sp. A	9	10
			sp. D		1
			sp. E		1
			sp. G		1
			sp. H	1	
			thione Banks, 1946	1	3
			utilis utilis (Cameron, 1891)	3	4
		Auplopus	*esmeralda* (Banks, 1925)	2	
			gaumeri Dreisbach, 1963	8	3
			sp. B	21	3
			sp. C		1
			sp. D		1
		Caliadurgus	*pruinosus* (Dreisbach, 1961)	1	6
		Dipogon	*moctezuma* Evans, 1999		1
		Minagenia	sp.	102	25
		Priocnemella	sp. B	4	6
			tabascoensis (Cameron, 1891)	1	4
		Priocnessus	sp.	5	1
	Pompilinae	*Agenioideus*	*birkmanni* (Banks, 1910)	5	10
		Allaporus	*smithianus* (Cameron, 1893)	71	44
		Anoplius	*amethystinus* (Fabricius, 1793)		2
			cuautemoc Evans, 1966		34
			decorus (Cameron, 1893)	3	
			sp.		6
		Aporinellus	*medianus* Banks, 1917		2
			yucatanensis (Cameron, 1893)		1
		Aporus	*chiapanus* Evans 1966	74	41
			concolor (Smith, 1860)		3
			idris idris (Cameron, 1897)	35	9
			notabilis (Smith, 1960)	15	7
		Epipompilus	*nigribasis* Banks, 1925	12	1
		Episyron	*cressoni* (Dewitz, 1881)		15
		Notocyphus	sp. B	3	44
			sp. C	4	
			violaceipennis Cameron, 1893	261	188
		Poecilopompilus	*mixtus* (Fabricius, 1794)		1
		Priochilus	*gracilis* Evans, 1966		6
			splendidulus (Fabricius, 1804)	2	
		Psorthaspis	*formosa* (Smith, 1862)	17	23
			variegata (Smith, 1862)	88	8
		Tachypompilus	*unicolor cerinus* Evans, 1966		1

Table S1 (cont.)

Family	Genus	Species	Forest	Farmland
Vespidae	*Agelaia*	*areata* (Say, 1837)	63	56
		centralis (Cameron, 1907)	6	6
	Brachygastra	*mellifica* (Say, 1837)		8
	Hypancistrocerus	sp.		1
	Mischocyttarus	*deceptus* (Fox, 1895)		3
		melanarius (Cameron, 1906)		5
		mexicanus mexicanus (Saussure, 1854)	6	5
	Pachodynerus	sp.		1
	Parachartergus	*apicalis* (Fabricius, 1804)		1
		aztecus Willink, 1959		1
	Polistes	*instabilis* Saussure, 1853		1
	Polybia	cf. *parvulina*	1	
		nigrina Richards, 1978	5	5
		occidentalis nigratella (du Buysson, 1905)	75	461
		rejecta (Fabricius, 1798)		1
		simillima Smith, 1862		1
	Santamenes	*novarae* (Saussure, 1867)		1
	Stenodynerus	*farias* (Saussure, 1857)	1	3
	Synoeca	*septentrionalis* Richards, 1978		3
